# Peroral direct digital cholangioscopy‐assisted retrieval of a sheared nasobiliary drainage tube through the overtube in a patient with surgically altered anatomy

**DOI:** 10.1111/den.14734

**Published:** 2023-12-19

**Authors:** Noriyuki Hirakawa, Katsuya Kitamura, Takao Itoi

**Affiliations:** ^1^ Department of Gastroenterology and Hepatology Tokyo Medical University Hachioji Medical Center Tokyo Japan; ^2^ Department of Gastroenterology and Hepatology Tokyo Medical University Tokyo Japan

## Abstract

Watch a video of this article.

## BRIEF EXPLANATION

Treatment of common bile duct stones or anastomotic stenosis is challenging in patients with surgically altered anatomy. The development of balloon endoscopy‐assisted endoscopic retrograde cholangiopancreatography has now enabled endoscopic treatment in these patients.^1^, ^2^ Here, we present a case in which a sheared tube remnant in a peripheral intrahepatic bile duct was successfully removed by peroral direct digital cholangioscopy‐assisted retrieval.^3^, ^4^, ^5^

**Figure 1:**
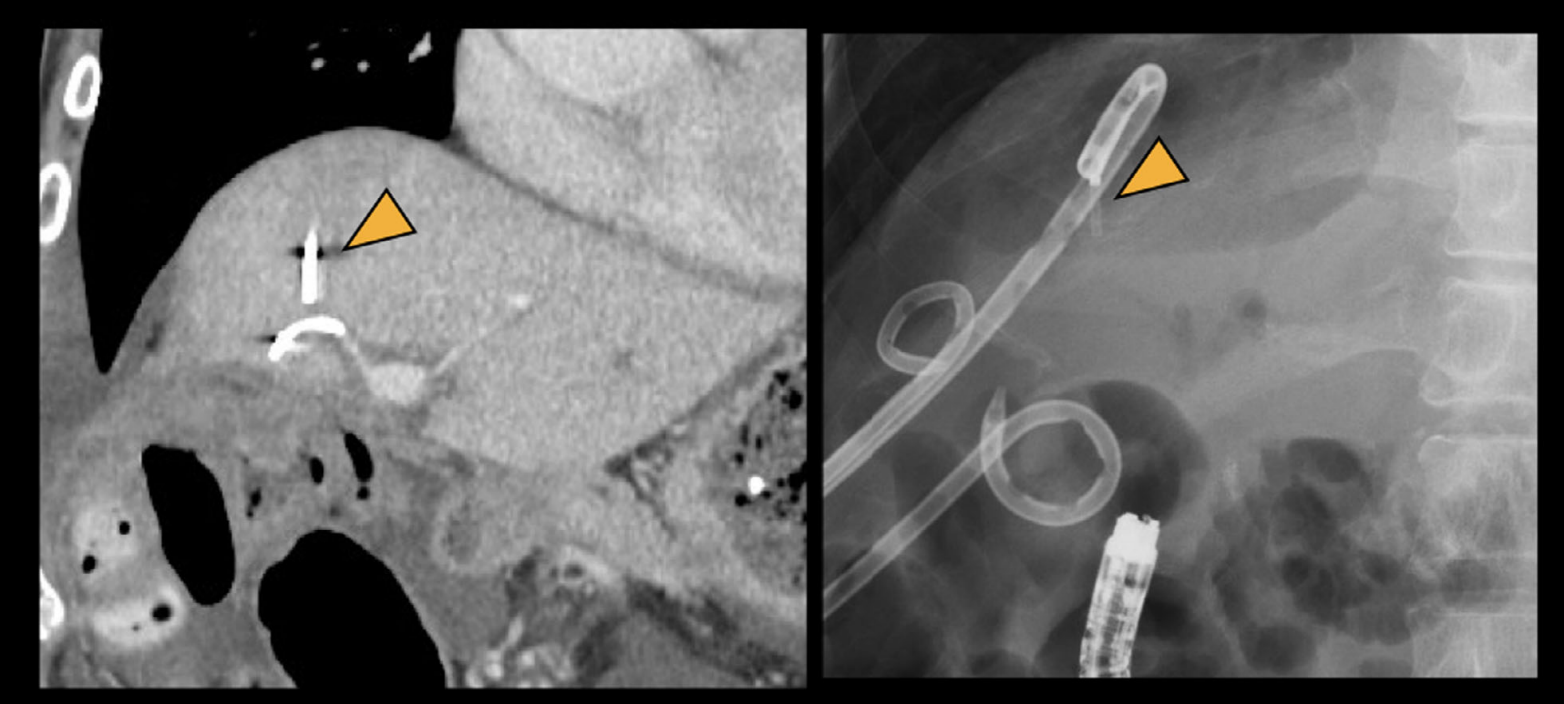
Computed tomography scan and radiograph showing a sheared nasobiliary drainage tube in an intrahepatic bile duct (arrowhead).

**Figure 2:**
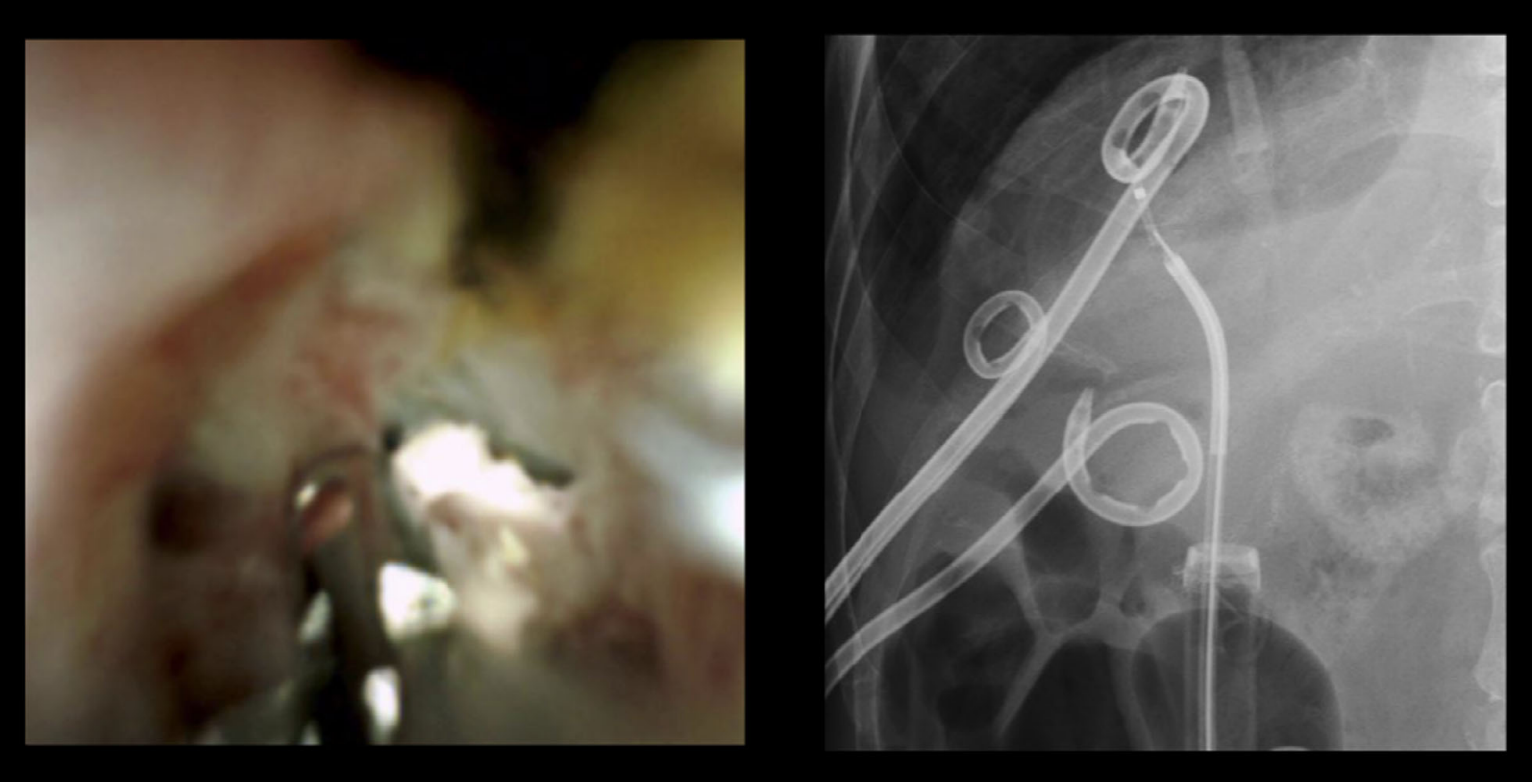
Grasping the tube with fine forceps under direct vision.

A 70‐year‐old man was admitted to our hospital for treatment of gallbladder cancer with colonic invasion. Extended cholecystectomy, pancreatoduodenectomy, and right hemicolectomy were performed. The patient was discharged but was later admitted to our hospital with a fever. A biliary fistula was diagnosed on abdominal computed tomography scan. Endoscopic nasobiliary drainage was performed by balloon endoscopy‐assisted endoscopic retrograde cholangiopancreatography using a short‐type single‐balloon endoscope (s‐SBE; SIF‐H290S; Olympus Medical, Tokyo, Japan). After magnetic resonance imaging was performed, the nasobiliary drainage tube was found to be sheared, possibly from the effect of magnetic resonance imaging on the metal at the end of the tube, leaving a remnant in an intrahepatic bile duct (Fig. 1). Retrieval using a basket catheter or balloon catheter was attempted but failed. In the next session, the s‐SBE was inserted to the anastomotic site, the overtube was advanced as deeply as possible for subsequent cholangioscope insertion, and a stiff guidewire was placed in the intrahepatic bile duct. The s‐SBE was removed, leaving the overtube and the guidewire in the bile duct. Then, a digital cholangioscope (SpyGlass DS Direct Visualization System, SpyDS; Boston Scientific, Natick, MA, USA) was inserted into the overtube and advanced via the guidewire to the bile duct where the sheared tube was located. The residual tube was successfully retrieved under direct vision by grasping the tube with fine forceps specially designed for oral biliary cholangioscopy (Fig. 2, Video S1).

Authors declare no conflict of interest for this article.

## Supporting information


**Video S1** Peroral direct digital cholangioscopy‐assisted retrieval of a sheared nasobiliary drainage tube through the overtube in a patient with surgically altered anatomy.
